# Enhancing Coordination Efficiency with Fuzzy Monte Carlo Uncertainty Analysis for Dual-Setting Directional Overcurrent Relays Amid Distributed Generation

**DOI:** 10.3390/s24134109

**Published:** 2024-06-25

**Authors:** Faraj Al-Bhadely, Aslan İnan

**Affiliations:** Department of Electrical Engineering, Yildiz Technical University, Istanbul 34220, Turkey; inan@yildiz.edu.tr

**Keywords:** overcurrent relay coordination, uncertainty, distributed generation, fuzzy Monte Carlo simulation, ALO

## Abstract

In the contemporary context of power network protection, acknowledging uncertainties in safeguarding recent power networks integrated with distributed generation (DG) is imperative to uphold the dependability, security, and efficiency of the grid amid the escalating integration of renewable energy sources and evolving operational conditions. This study delves into the optimization of relay settings within distribution networks, presenting a novel approach aimed at augmenting coordination while accounting for the dynamic presence of DG resources and the uncertainties inherent in their generation outputs and load consumption—factors previously overlooked in existing research. Departing from conventional methodologies, the study proposes a dual-setting characteristic for directional overcurrent relays (DOCRs). Initially, a meticulous modeling of a power network featuring distributed generation is undertaken, integrating Weibull probability functions for each resource to capture their probabilistic behavior. Subsequently, the second stage employs the fuzzy Monte Carlo method to address generation and consumption uncertainties. The optimization conundrum is addressed using the ant lion optimizer (ALO) algorithm in the MATLAB environment. This thorough analysis was conducted on IEEE 14-bus and IEEE 30-bus power distribution systems, showcasing a notable reduction in the total DOCR operating time compared to conventional characteristics. The proposed characteristic not only achieves resilient coordination across a spectrum of uncertainties in both distributed generation outputs and load consumption, but also strengthens the resilience of distribution networks overall.

## 1. Introduction

### 1.1. Importance

In the dynamic realm of power distribution systems, the integration of distributed generation resources presents a distinctive array of hurdles in ensuring the dependable and effective protection of power networks. Coordinating directional overcurrent relays (DOCRs) become increasingly intricate when faced with uncertainties arising from both distributed generation (DG) outputs and load consumption patterns. Conventionally, relay configurations have been established without considering the dynamic presence of DG sources or fluctuations in their performance, thereby constraining the adaptability of the distribution networks. Traditional DOCRs are typically set to trigger at a fixed current level and direction, predicated on predetermined settings that are primarily suited to standard operating conditions. Nevertheless, the increasing incorporation of DG into power systems has introduced new challenges for protection and coordination. DG sources such as solar and wind power, exhibit variability in their output due to factors such as weather conditions and fluctuations in demand. This variability can result in shifts in fault currents and locations within the power system, complicating the maintenance of effective coordination using conventional single-setting DOCRs. To address these challenges, recent research has concentrated on the development of dual-setting DOCRs, which provide greater flexibility and adaptability in coordinating protection schemes in the presence of DG. Dual-setting relays enable a single relay to function in both the forward/primary and reverse/backup directions, simultaneously. This innovative programming approach augments relay operation efficiency by reducing the total relay tripping time. In light of these advancements, the following section presents a literature review encompassing recent modern research in this area.

### 1.2. Literature Review

Traditional directional overcurrent relays (DOCRs) have long been the cornerstone of power system protection strategies, offering fixed parameters for pickup currents, time delays, and directional features. However, as power systems undergo transformations with the integration of distributed generation (DG) and encounter heightened uncertainties, the shortcomings of single-setting relays have become apparent. Early investigations into traditional DOCRs primarily focused on optimizing relay configurations for coordination and fault detection under stable operational conditions. Pioneering research [1,2,3,4,5] underscored the significance of precisely configuring relay parameters to ensure efficient coordination and mitigate the risk of erroneous operations. Similarly, other studies [6,7] shed light on the complexities of coordinating protection schemes in systems with extensive DG penetration, proposing heuristic-driven methodologies to bolster coordination reliability. However, these endeavors have yet to present a comprehensive framework for modeling uncertainties in distribution power networks. As power systems transition towards more decentralized and dynamic architectures, the necessity for adaptive protection schemes becomes paramount [8]. Dual-setting DOCRs have emerged as a promising solution for addressing the limitations of single-setting relays and enhancing protection coordination in modern power systems [9]. These dual-setting relays offer flexibility to independently adjust the pickup currents and time delays for each directional setting, thereby enabling improved fault detection and discrimination across various operating scenarios [10]. Recent advancements in dual-setting DOCRs have been spurred by the incorporation of advanced optimization techniques and uncertainty analysis. Several studies [11,12,13] introduced optimization algorithms tailored for dual-setting relays, leveraging methodologies such as genetic algorithms and particle swarm optimization to identify optimal relay settings while considering system constraints and coordination requisites. These methodologies demonstrate enhanced coordination capabilities. In addition, a scheme based on the overcurrent theory using a supervisory system with communication capabilities was developed to re-evaluate the coordination for every topological change in the power system [14]. Furthermore, the behavior of overcurrent relays was studied, concentrating on operating speed and relay selectivity to ensure proper coordination of the protection system [15]. Alternatively, the manta ray foraging optimization approach was utilized to enhance overcurrent relay (OCR) configurations and maintain coordination tolerances within relay pairs [16], while simulated annealing–linear programming was used to obtain optimal OCR coordination [1]. Moreover, a comprehensive analysis of the selectivity challenges in OCR design based on statistical data was carried out [17]. Furthermore, the tripping characteristics of OCR relays, both standard and non-standard, were investigated using the tug-of-war optimization algorithm and the charged system search algorithm [18]. Consequently, to address the increased short-circuit currents arising from DG integration, an online communication system with multi-setting relays developed from offline experiments was incorporated [19]. In addition, a protective system based on communication linkages between OCRs with ideal standard features placed on both sides of the distribution lines and protection zones was suggested [20]. Furthermore, modern numerical relays with multifunctional data storage, communication, and signal processing functions were used to optimize relay settings while considering the various operational scenarios related to DG resources. An advanced two-level algorithm intended to optimize protective relay parameters by integrating various operating scenarios within communication systems was also introduced [21]. In another study, relay working times were determined using only the local voltage magnitude [22]. In a further study, a novel OCR-based area protection scheme designed especially for distribution networks with significant DG source penetration was proposed [23]. Additionally, wind speed and direction predictions were employed to facilitate online relay settings, initially determined offline using optimization techniques [24]. Furthermore, a unique non-communication-based time–current–voltage dual-setting directional overcurrent prevention strategy designed specifically for distribution systems with DG integration was also introduced [25]. The sine–cosine technique was used to overcome optimal coordination issues associated with directional OCRs [26]. In a further work, network topology groups and ideal relay settings were discussed concurrently [27]. Furthermore, support vector machines were used as a classifier to establish predefined trip times for OCRs [28]. Moreover, a multi-agent system-based hierarchical protection approach was used to provide a comprehensive protection strategy, with higher levels of updated relay settings and lower ones concentrating on fault clearance and protection coordination [29]. Ultimately, to increase the versatility of protection methods, user-defined dual-setting OCRs with hybrid time current-voltage characteristics were designed [30]. Numerous studies have investigated the protection of optimized microgrids (MGs) and smart grids (SGs), such as those referenced in [31,32]. However, their focus was primarily on the foundational network configuration and selectivity constraints. This myopic approach risks selectivity constraints that breach alternate topologies. Moreover, the absence of a comprehensive modeling strategy for uncertainties poses significant challenges in real-world applications. In light of these limitations, some scholars, as exemplified in [33,34], have explored protection coordination across diverse grid configurations, while protecting MGs/SGs with a singular setting group sans additional communication infrastructure for various configurations. However, while this is convenient and practical, it may impede protection speed due to multifarious constraints and a restricted search space for DOCRs. Entering the concept of adaptive protection is a pragmatic avenue to address concerns in MG/SG protection across varying operating conditions and topologies. Nonetheless, the implementation of adaptive protection systems necessitates extensive telecommunication links and introduces uncertainty into their operations [34]. However, dependable communication-aided adaptive schemes have significant advantages. Numerous research endeavors have scrutinized adaptive protective schemes for SGs/MGs. For instance, the authors of [35] introduced an adaptive and robust protection mechanism that accounts for the power quality characteristics and voltage indicators. Similarly, the authors of [36] reported adaptive protection considering the stability constraints of distributed generations (DGs), whereas [37] focused on reducing coordination optimization constraints. However, it is crucial to optimize the current and time settings of OCRs while overlooking optimization of the characteristic curves. This oversight, coupled with the absence of a comprehensive method to address uncertainties, renders these methods practical, yet potentially less efficient in real-world scenarios. In active distribution networks (ADNs), SGs, and MGs, uncertainties stemming from load and generation units can lead to protection miscoordination. To address this challenge, a study [38] proposed clustering different network topologies into a limited set, facilitating the determination of optimal relay settings for each cluster. This approach, simultaneously tackles setting groups and optimal network topology clusters, while also investigating the impact of relay characteristics on reducing operating times. Despite these advancements, the number of available setting groups remains significantly lower than the number of potential configurations, limiting multiple-setting group-based protective schemes to a handful of topologies and operation modes. In [39], a pioneering setting-group-based scheme for protecting networked MGs employing DOCRs was introduced. This scheme ensures adequate protection across all MGs under various interconnections with the utility grid. However, while [39] offers a model for categorizing MG interconnections into distinct groups and determining optimal relay settings, it falls short in accommodating diverse states of uncertainties in the protection scheme. However, these studies have yet to present a practical and comprehensive scheme for modeling uncertainties in protection settings, which could potentially impact the performance and reliability compared to traditional methods. Reference [40] introduced a methodology aimed at addressing uncertainty in demands and DGs within distribution systems through Monte Carlo simulation, a statistical technique that generates multiple random samples of uncertain parameters to analyze system behavior and understand the impacts of uncertainties on system performance. However, the neglect of the fuzzification of relay inputs in this research raises concerns about the efficiency and accuracy of the proposed scheme. The incorporation of uncertainty analysis using fuzzy Monte Carlo techniques has further bolstered the capabilities of dual-setting DOCRs to mitigate uncertainties stemming from DG integration and load variations, aspects overlooked in previous state-of-the-art research. Although significant strides have been made in the development and application of dual-setting DOCRs, challenges persist, including the lack of standardized methodologies for relay parameter optimization and coordination algorithms. Future research endeavors should prioritize addressing these challenges to propel power system protection toward meet the evolving requirements of modern power systems.

### 1.3. Contribution

The integration of dual-Setting directional overcurrent relays (DOCRs) with uncertainty analysis introduces a pioneering methodology that seamlessly merges DOCRs with uncertainty modeling utilizing fuzzy Monte Carlo methods, which incorporates fuzzy Monte Carlo simulation to effectively model and address uncertainties stemming from factors such as load variations, intermittent renewable energy generation, and equipment failures. By doing so, it enhances the adaptability of relay coordination to fluctuating operating conditions, consequently bolstering the system reliability and performance. Moreover, the proposed fuzzy Monte Carlo simulation possesses several distinctive capabilities that have been overlooked in previous state-of-the-art studies. The primary contributions of our study can be succinctly outlined as follows:

- Capturing Imprecise Information: Unlike conventional probabilistic methods that rely on precise probability distributions, fuzzy logic enables the representation of imprecise or vague information inherent in real-world systems. This feature proves invaluable in scenarios where obtaining precise data is challenging.
- Flexibility in Modeling: Fuzzy Monte Carlo offers unparalleled flexibility in modeling uncertainties by accommodating expert knowledge or subjective assessments. This adaptability allows for the inclusion of qualitative factors, thereby enhancing the realism and accuracy of the uncertainty model. Fuzzy logic excels in handling the nonlinear and complex relationships often encountered in engineering and decision-making contexts.
- Robustness to Data Limitations: In situations where data availability is limited or uncertain, fuzzy Monte Carlo remains robust by incorporating expert knowledge or heuristic information. This resilience to data limitations renders fuzzy Monte Carlo indispensable in scenarios where empirical data may be scarce or unreliable.
- Integration with Monte Carlo Simulation: By integrating fuzzy logic with Monte Carlo simulation, fuzzy Monte Carlo harnesses the strengths of both approaches. This integration ensures the efficient generation of probabilistic outcomes while capturing qualitative aspects of uncertainty.

Additionally, this study contributes to the practical application of uncertainty modeling by employing a Weibull distribution to accurately depict the variability and unpredictability of DG power generation and demand in real-world scenarios. Furthermore, a Monte Carlo simulation approach with a fixed number of iterations was utilized to address uncertainties in the input variables, such as DG output and load demand. This simulation technique guarantees the accurate and efficient sampling of uncertain variables, leading to enhanced convergence and a comprehensive understanding of the potential outcomes and behaviors of the power system under varying conditions. Finally, the proposed methodology enhances the efficiency and resilience of protection schemes by offering insights into the impact of uncertainties on protection coordination and presenting practical solutions for their mitigation. Conventional relay coordination typically involves fixed relay settings based on predetermined system parameters. It is proposed that dual-setting relay coordination allows for the dynamic adjustment of relay settings in response to changing system conditions or events. This dynamic adaptation enhances the flexibility and robustness of the coordination scheme, enabling better fault detection and isolation, particularly in systems with a high DG penetration and variable generation patterns.

### 1.4. Organization

The rest of the paper is organized as follows. Section 2 discusses the conceptual framework and the probabilistic model underpinning the fuzzy Monte Carlo method. In Section 3, we detail the mathematical formulation of our proposed technique. Section 4 presents the simulation results and a thorough discussion of these findings. Lastly, Section 5 summarizes the key conclusions drawn from the study.

## 2. Probabilistic Model of Fuzzy Monte Carlo

**Figure 1:**
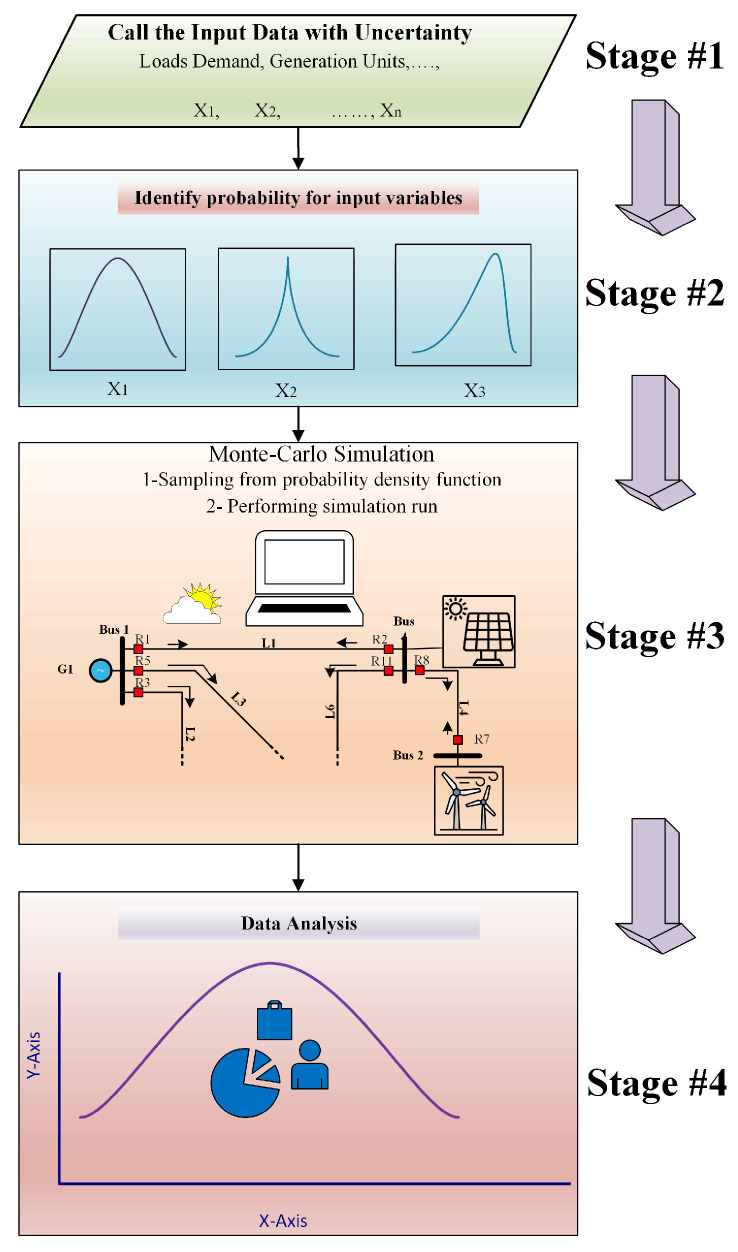
Conceptual Model of Proposed Protection scheme Using Fuzzy Monte Carlo Probabilistic Model.

The probabilistic model of fuzzy Monte Carlo (PFMC) is a pivotal approach in the realm of power system protection that directly handles the dual challenges posed by randomness and vagueness. In essence, probabilistic modeling takes the center stage, offering a method to encapsulate uncertainties stemming from randomness. Here, probability distributions, including Weibull, exponential, and log-normal distributions, serve as robust tools to depict the uncertainties linked to equipment failure rates and fault occurrence probabilities, as elucidated subsequently. In this vein, this study amalgamates both probabilistic and fuzzy logic methodologies, crafting a holistic framework tailored to model uncertainties in power system protection. Furthermore, the use of Monte Carlo simulations serves as a cornerstone, facilitating the dissemination of uncertainties throughout the protection system, thereby evaluating its efficacy across diverse operational scenarios. Random samples are drawn from the probability distributions of the input parameters, whereas fuzzy inference systems process fuzzy inputs adeptly, yielding fuzzy outputs. Through the simulation of myriad scenarios, the Monte Carlo methodology provides a thorough assessment of the protection system’s reliability, resilience, and effectiveness in navigating risks under uncertain conditions. Illustrating the conceptual underpinning of the proposed protection scheme, Figure 1 offers a visual depiction outlining the framework’s key components and their interrelationships.

### 2.1. Calculation of Weibull Distribution Parameters

Robust modeling techniques are employed to address the variability and unpredictability inherent in power system parameters such as load demands and DG output. This section outlines the approach for modeling uncertain variables and provides insights into the methodologies utilized. The DG output pattern follows a Weibull probability density function (PDF) [41], as shown in Equation (1).

(1) $$f\left(x\right)=\frac{\beta }{\alpha^{\beta }}\;x^{\beta -1}\;\exp\left[-{\left(\frac{x}{\alpha }\right)}^{\beta }\right]$$

The uncertainty associated with the load can be represented using a normal PDF, as shown in Equation (2).

(2) $$f\left(x\right)=\frac{1}{\sigma \sqrt{2\pi }}\;e^{-\frac{{\left(x-\mu \right)}^{2}}{2\sigma^{2}}}$$

In this method, *y* = *f*(*x*) is regarded as a multivariate function, where x=x1,x2,x3,…,xn represents a vector of uncertain random input variables. It is assumed that the PDFs of the input variables are known. The objective was to derive the PDF of *y*, the output variable.

### 2.2. Monte Carlo Simulation with PDFs

In addition to calculating the Weibull distribution parameters, the probabilistic modeling method incorporates uncertainty modeling using the fuzzy Monte Carlo method. A straightforward approach with a fixed number of iterations was adopted to perform the Monte Carlo simulation. This technique facilitates the accurate and efficient sampling of uncertain variables, resulting in improved convergence and reliability of the analysis. In each iteration of the Monte Carlo simulation, the values for the uncertain input variables were sampled from their respective probability distributions or fuzzy sets. Generating a large number of samples allows a comprehensive understanding of the potential outcomes and behaviors of the system under varying conditions. Over the past decade, the increasing penetration of DG has fundamentally changed the operation and protection of distribution networks. In this study, the previous research on single-setting DOCRs was extended by developing a methodology for optimal coordination using dual-setting DOCRs. This methodology involves the integration of advanced optimization techniques with uncertainty analysis to ensure robust and reliable protection coordination in the presence of a DG source. Incorporating uncertainty modeling using fuzzy Monte Carlo methods allows accounting for the variability and unpredictability associated with DG output and system conditions. This allows the identification of optimal relay settings that are adaptive to changing operating conditions and can mitigate the effects of uncertainties on protection coordination. Overall, this work builds upon the foundation of previous research on single-setting DOCRs and advances the state-of-the-art in power system protection by introducing dual-setting DOCRs with enhanced flexibility, adaptability, and robustness in coordinating protection schemes in the presence of distributed generation and uncertainty. The contribution of DG sources to faults depends on factors such as the generating capacity of the DG source (size of the DG source), the distance from the DG source to the fault location and the type of DG source. To represent the variability and uncertainty of (DG) power generation and demand in real world scenarios, we used a Weibull distribution. In addition, a simple Monte Carlo simulation approach with a fixed number of iterations was employed. This technique provides an accurate and efficient sampling of uncertain variables, leading to better convergence. A Monte Carlo simulation was performed to account for the uncertainty in the input variables. In each simulation iteration, the sample values for the uncertain input variables are obtained from their respective probability distributions or fuzzy sets.

## 3. Problem Formulation

The operating time of a DOCR typically inversely correlates with the short-circuit current it experiences, a relationship that has been thoroughly explored in numerous studies on protection systems. The time–current characteristic of the relay is generally described as in Equation (3) [42].

(3) $$T_{ik}=\frac{{\mathrm{TMS}}_{i}\;\times A}{{\left(\frac{I_{F,ik}}{{\mathrm{PS}}_{i}}\right)}^{B}-1}$$

where *i* denotes the relay identifier, and *k* signifies the fault location identifier. The constants *A* and *B*, which are often adjusted to 0.14 and 0.02, respectively, depend on the kind of OCR. The relay fault current is denoted by $I_{F,ik}$, while $PS_{i}$ represents the relay pickup current.

### 3.1. Modeling Dual-Setting DOCR Characteristics

Dual-setting directional overcurrent relays (DS-DOCRs) represent a significant leap forward in protective relay technology. These relays, designed to operate in both the forward and reverse directions, offer primary and backup protection functionalities. In contrast to conventional DOCRs restricted to one direction, DS-DOCRs provide bidirectional fault detection capabilities. Leveraging advanced mechanisms, DS-DOCRs are adept at detecting faults in both directions and triggering responses based on pre-defined time and pickup settings.

**Primary Protection:** This denotes the principal function of the relay, triggered when the fault current flows in the forward direction. The settings for primary protection are designated as $TMS_{fw}$ and $PS_{fw}$, where $TMS_{fw}$ represents the time multiplier setting, and $PS_{fw}$ represents the pickup setting.

**Backup Protection:** This serves as a secondary protection function of the relay and is activated when the fault current flows in the reverse direction. The settings for backup protection are indicated as $TMS_{rv}$ and $PS_{rv}$, where $TMS_{rv}$ represents the time multiplier setting and $PS_{rv}$ represents the pickup setting.

**Figure 2:**
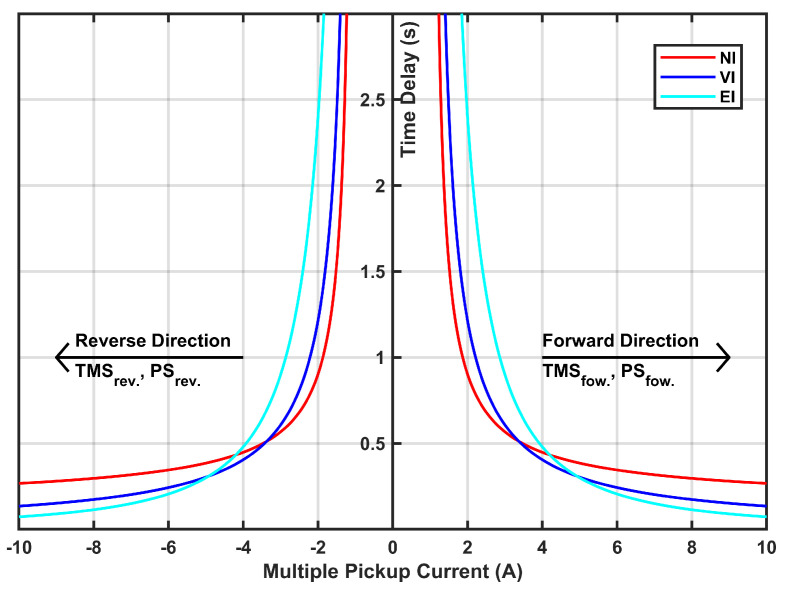
Time–current characteristics for the dual-setting DOCR.

**Time–Current Characteristic:** This refers to a graphical representation illustrating how the relay responds to various fault currents over time. Figure 2 illustrates the time–current characteristic curve of the relay, depicting its response for both primary and backup protection operations.

**Figure 3:**
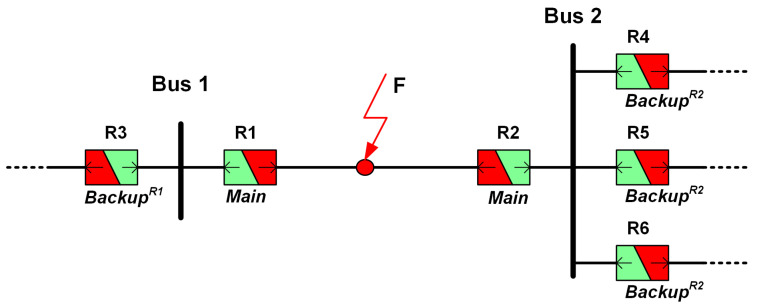
Protection with dual-setting directional relays.

Figure 3 illustrates an example of a dual-setting directional overcurrent relay equipped with the relay characteristics depicted in the same figure. Each relay is represented by two arrows, indicating the two directions of the fault current flow for which the relay can operate. The red-colored parts of the DOCRs represents the forward-directed protection section, while the green-colored parts indicates the reverse-directed protection section. In the event of a fault occurring at point F, the relay configurations are as follows: $R_{3}$ serves as the backup relay for $R_{1}$, and $R_{4}$, $R_{5}$, and $R_{6}$ function as backup relays for $R_{2}$. In such scenarios, $R_{3}$ utilizes settings associated with reverse operation ($TMS_{rv3}$ and $PS_{rv3}$), whereas $R_{1}$ employs settings linked with forward operation ($TMS_{fw1}$ and $PS_{fw1}$), and similarly for other relays. This configuration ensures comprehensive protection coverage for fault occurrences in both forward and reverse directions.

**Figure 4:**
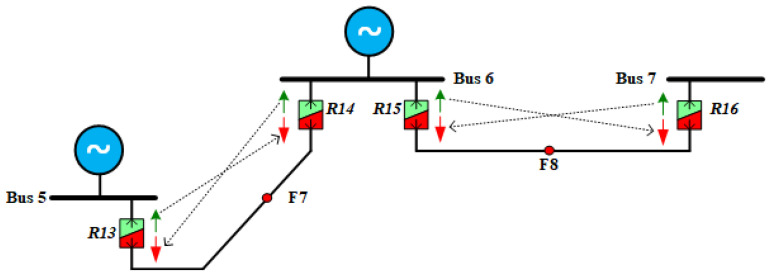
Illustration of a segment from the test system, emphasizing the communication approach employed.

Previous research, e.g., [43], has highlighted the challenges in ensuring proper coordination between relays with dual settings. Therefore, the proposed scheme emphasizes the importance of efficient communication even in low-bandwidth scenarios to ensure effective coordination for backup operations, as discussed in [44]. Figure 4 illustrates a section of the test system, emphasizing the communication strategy employed between the dual-setting relays. The essence of this strategy lies in establishing communication between the relays operating in the reverse and forward directions along the same lines. This communication ensures that the reverse direction characteristic of each relay communicates with the forward direction characteristic of the adjacent relay, effectively blocking its operation when necessary. By implementing this communication strategy across all relays in the system, we aim to prevent scenarios such as the unintended operation of a relay (e.g., R13 in the forward direction) for a fault at a specific location (e.g., F8). This proactive approach not only maintains the achieved reduction in fault isolation time but also ensures proper protection coordination among the relays. It is important to note that the communication mechanism primarily applies to the backup relay operations. Therefore, in the event of a communication failure or delay, the primary relays remain unaffected, and the protection scheme can still isolate faults in a timely manner. For instance, in the case of a fault at F8, the R15’s forward characteristic will operate and isolate the fault regardless of the communication link’s status. Even if R15 fails to operate because of a malfunction and the communication link is lost, R13 will still operate before relay R14, guaranteeing fault isolation, albeit with temporary coordination discrepancies.

### 3.2. Objective Function

Due to the presence of fault currents flowing in both the forward and reverse directions, certain manufacturers have proposed dual-setting DOCRs to respond differently in each direction. In [34], the following objective function is present in Equation (4):

(4) $$\mathrm{OBJ}=\min\left[\sum_{k=1}^{M}\left(\sum_{i=1}^{N^{fw}}T_{i,k}^{fw}+\sum_{j=1}^{N^{rv}}T_{j,k}^{rv}\right)\right]\;\forall \left(i,j\right)\in \omega$$

In this context, ω represents the set of relay P/B pairs and *N* denotes the total number of relays, while *M* indicates the total number of fault locations along all lines. The terms $T_{i,k}^{fw}$, and $T_{j,k}^{rv}$ correspond to the tripping times of relays *i*, and *j* for a fault at location *k* during forward and reverse operations, respectively, as described by Equations (5) and (6):

(5) $$T_{i,k}^{fw}=\frac{{\mathrm{TMS}}_{i}^{fw}\;\times A}{\left({\left(\frac{I_{F,i}^{fw}}{{\mathrm{PS}}_{i}^{fw}}\right)}^{B}-1\right)}$$

(6) $$T_{j,k}^{rv}=\frac{{\mathrm{TMS}}_{j}^{rv}\;\times A}{\left({\left(\frac{I_{F,j}^{rv}}{{\mathrm{PS}}_{j}^{rv}}\right)}^{B}-1\right)}$$

In this context, $TMS_{i}^{fw}$ and $TMS_{j}^{rv}$ are the time multiplier settings for relays *i* and *j* in the forward and reverse directions, respectively. Similarly, $PS_{i}^{fw}$ and $PS_{j}^{rv}$ denote the plug settings of relays *i* and *j* for forward and reverse operations, respectively. The fault current at location *k*, passing through relay *i* in the forward direction, is represented as $I_{F,i}^{fw}$. Likewise, $I_{F,j}^{rv}$ indicates the fault current passing through relay *j* in the reverse direction due to a fault at location *k*. The coordination constraints, which must be met to solve the protection coordination problem, can be expressed by Equation (7).

(7) $$T_{j,k}^{rv}-T_{i,k}^{fw}\geq \mathrm{CTI}\;\forall i,j,k$$

The coordination time interval CTI represents the minimum time difference between the operation of primary and backup relays. Typically, the CTI ranges from 0.2 to 0.5 s; in this study, it was set to 0.2 s. Furthermore, there are upper and lower bounds on the relay settings, defined by Equations (8) and (9), respectively.

(8) $${\mathrm{PS}}_{\min}\leq \left({\mathrm{PS}}_{i}^{\mathrm{fw}},{\mathrm{PS}}_{j}^{\mathrm{rv}}\right)\leq {\mathrm{PS}}_{\max}$$

(9) $${\mathrm{TMS}}_{\min}\leq \left({\mathrm{TMS}}_{i}^{\mathrm{fw}},{\mathrm{TMS}}_{j}^{\mathrm{rv}}\right)\leq {\mathrm{TMS}}_{\max}$$

In this context, $PS_{\min}$ and $PS_{\max}$ denote the lower and upper bounds, respectively, for relay *i*’s plug setting. Similarly, $TMS_{\min}$ and $TMS_{\max}$ represent the lower and upper bounds for the time multiplier setting for relay *i*.

### 3.3. ALO Optimization Approach for Addressing Coordination Issues

This study introduces an optimization algorithm referred to as the ant lion optimizer (ALO), which is meticulously crafted to effectively address coordination challenges. In particular, the ALO method is employed to determine the optimal values for both the pickup current and Time Multiplier Setting (TMS) of dual-setting DOCRs deployed within distribution systems integrated with DG sources [45]. The ALO used in this study employs an initial population of 250 antlions. As a population-based metaheuristic algorithm, ALO operates on a matrix of multiple antlion and ant individuals. The number of antlions in the initial population was set to 250, which is within the common range of 20–500 individuals used for population-based optimization algorithms. This population size of antlions, along with the population of ants, forms the basis for the cooperative hunting mechanism at the core of the ALO optimization approach. The operational steps of the proposed ALO algorithm, designed to minimize the operating time of the dual-setting DOCRs, are outlined as follows:

**Step 1: Input Acquisition and System Data Analysis:**
  - Acquire input parameters, encompassing distribution system data and relay specifications.
  - Establish primary/backup relay pairs based on gathered data.
  - Calculate both the full load and short circuit currents for each relay to ascertain their operational conditions.
**Step 2: Initializing Population:**
  - Generate initial populations of both antlions and ants, distributing them randomly while adhering to the defined upper and lower parameter constraints.
**Step 3: Evaluating Fitness Function:**
  - Evaluate the fitness function, representing the total relay operating time, for all ants and antlions by employing the objective function index for assessment.
**Step 4: Identifying Elite Antlions:**
  - Identify elite antlion solutions by discerning the top-performing candidates.
**Step 5: Updating Positions:**
  - Adjust parameters to steer ants toward the central point of attraction, represented by antlion.
**Step 6: Generating Random Walk:**
  - Devise a random-walk mechanism to normalize the movement patterns of both ants and antlions.
**Step 7: Adjusting Positions for Antlions:**
  - Update the positions of antlions utilizing a predefined formula to refine their spatial locations.
**Step 8: Evaluating Solutions:**
  - Assess the best solution and substitute the antlion with the corresponding ant if it outperforms an elite candidate.
**Step 9: Storage and Termination:**
  - If the maximum iterations are attained, store and present the optimal results achieved through the algorithm.

**Figure 5:**
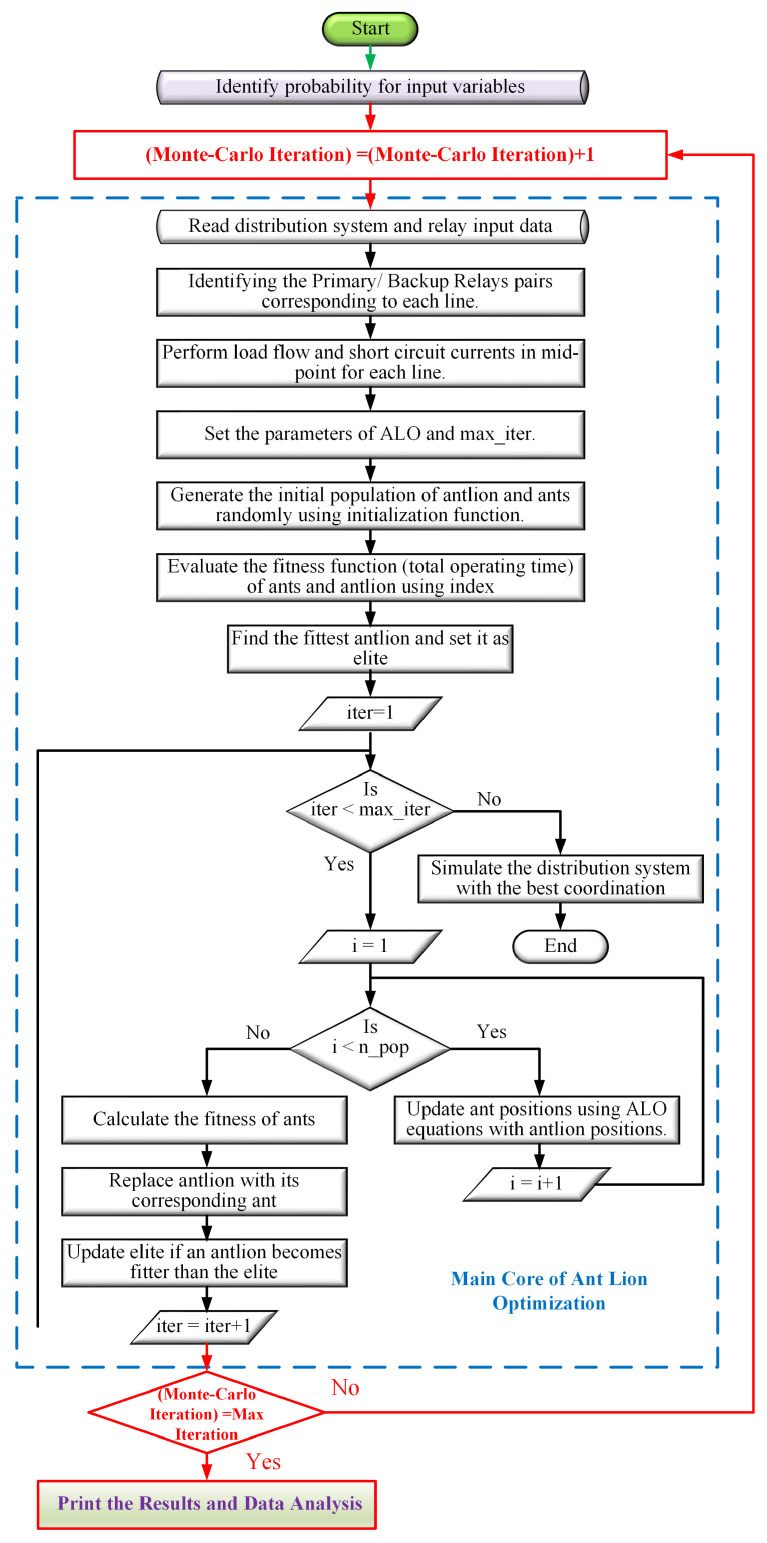
Flowchart of the ant lion optimization (ALO) algorithm.

A flowchart detailing the operational steps of the ALO algorithm is presented in Figure 5.

## 4. Case Study and Results

### 4.1. Case 1: IEEE 14-Bus System

**Figure 6:**
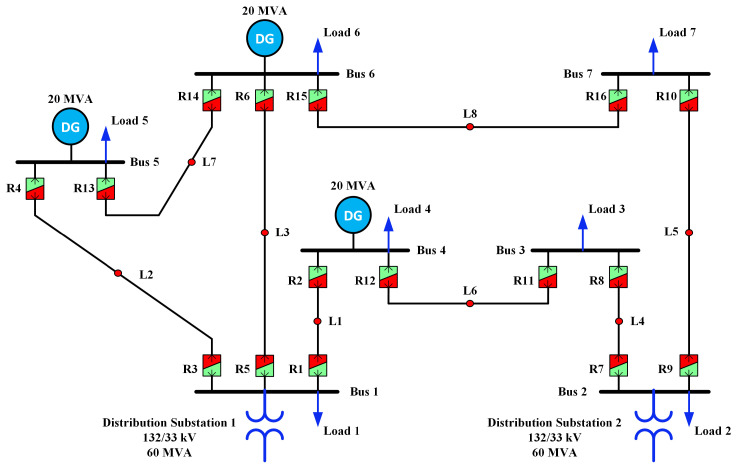
Modified distribution portion of the IEEE 14-bus system.

In this case study, the adapted distribution section of the IEEE-14 bus system, representing a 7-bus microgrid setup as illustrated in Figure 6, was utilized to analyze the proposed protection method. This configuration includes three (DG sources with a capacity of 20 MVA each linked to buses B4, B5, and B6. Moreover, the 7-bus microgrid system is connected to the sub-transmission network via buses B1 and B2, each with a generation capacity of 60 MVA. Further specifications of the test system can be found in Ref. [46]. This test setup comprised eight lines, protected by 16 dual-setting DOCRs positioned at both line ends. There are 22 relay pairs ($RP_{1}$–$RP_{22}$). Specifics of the number of primary and backup relays are outlined in Table 1.

#### Simulation Result, Using ALO Algorithm

In this scenario, the metaheuristic ALO algorithm was applied to attain the optimal coordination of dual-setting DOCRs within the test network. A comprehensive, step-by-step elucidation of the implemented algorithm is as follows.

**Step 1: Optimal Setting Without Considering Uncertainty:**

The fault current values recorded via the primary and backup relays for the distribution part of the IEEE-14-bus system are presented in Table 2. The operating times of the primary and backup relay pairs and their associated CTI values are listed in Table 3. The optimal relay setting values for the dual-setting DOCRs are presented in Table 4.

**Step 2: Optimal Relay Setting Considering Uncertainty:**

The stochastic nature of DG sources and electrical loads introduces uncertainties that profoundly affect the operation of microgrids and distribution networks. Failure to address these uncertainties can result in unrealistic and inaccurate modeling outcomes. Consequently, in this phase, the uncertainties linked to the DG sources and electrical loads were taken into account to effectively optimize the relay settings. To address the challenges posed by uncertainty, a probabilistic analysis is seamlessly integrated into the relay coordination process. By factoring in the variability in DG output power and load demand, the approach ensures that relay settings exhibit robustness and adaptability to a spectrum of operating conditions. This method not only bolsters the reliability and resilience of the distribution network but also fortifies it against unforeseeable fluctuations in both power generation and consumption. The subsequent sections provide a detailed overview of the input data used in the analysis, with a specific focus on the uncertainties associated with the load demand and output power of DG sources within the IEEE 14-bus test system.

**Figure 7:**
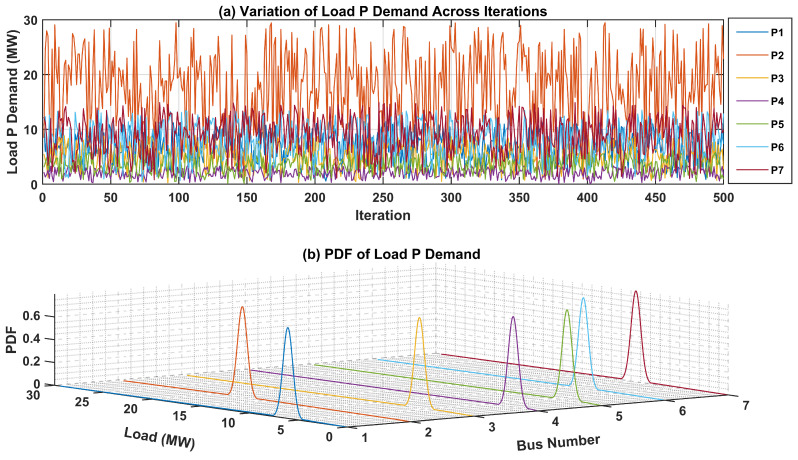
Variation in and PDF of Active Power Load Demand in IEEE-14 Bus system.

**Figure 8:**
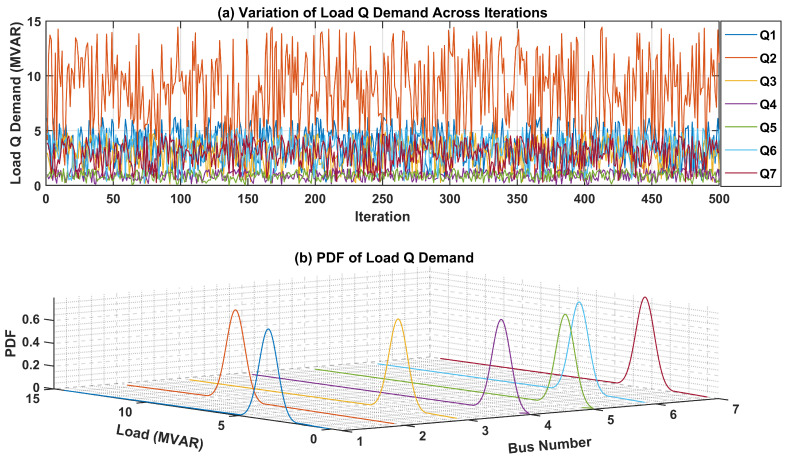
Variation in and PDF of Reactive Power Load Demand in IEEE-14 Bus system.

**Figure 9:**
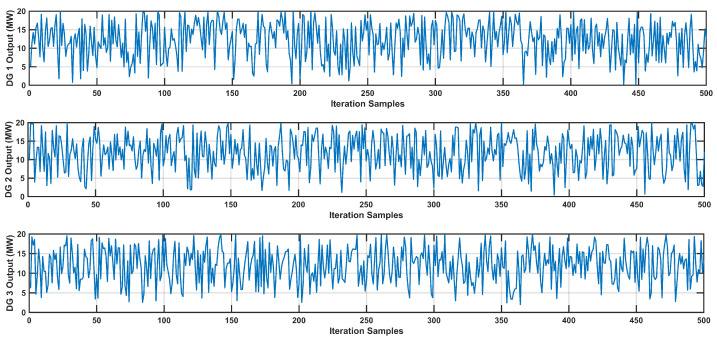
Variation in DG Outputs Across MCS Iterations in IEEE-14 Bus system.

1. **Load Uncertainties**
  The uncertainties surrounding the load demand at each bus within the test system are critical for precise modeling and analysis. To visually represent these uncertainties, PDFs for both active and reactive power of load demands at each bus are presented. In Figure 7a, the fluctuation in active power load demand across numerous iterations is showcased, offering insights into its variability over time. Additionally, the 3D plot in Figure 7b illustrates the PDF of the active power load demand for each bus. Each curve in this plot represents the PDF of active power load demand at a specific bus, offering a graphical representation of the uncertainties associated with the active power load demand.
  Likewise, Figure 8a portrays the fluctuation in the reactive power load demand across various iterations, providing insight into its variability over time. The 3D plot in Figure 8b presents the PDF of the reactive power load demand for each bus in the test network. These graphical depictions serve to elucidate the uncertainties inherent in load demand across different buses within the network, thereby facilitating more robust modeling and analysis processes.
2. **Distributed Generation Source Output Power Uncertainties**
  In addition to load uncertainties, uncertainties exist in the output power of DG sources. Figure 9 illustrates the variation in DG source output power across multiple iterations, offering insights into the variability of DG source power output. Each subplot in the figure represents the output power of a specific DG source throughout the iterations, effectively showcasing the uncertainties associated with DG source power generation.
3. **Monte Carlo Simulation**
  One of the most prevalent and accurate stochastic methods used in this study is Monte Carlo simulation (MCS), where the sample sizes are typically set to $N_{s}=500$. The MCS is an iterative process that encompasses the following steps:
  - The behavior of the power system for each sample scenario was simulated by considering the generated samples of uncertain parameters.
  - Short-circuit analysis was conducted to ascertain the fault currents in the system for each sample scenario and store these fault currents in a cell array corresponding to each scenario.
  - An optimization algorithm (such as ALO) is utilized to determine the optimal settings for relay coordination based on the fault currents obtained from short circuit analysis. The objective is to minimize the operating time while ensuring proper relay coordination.
  - After obtaining the optimal settings for relay coordination for each sample scenario, the PDFs for the TMS and PS parameters were obtained for both forward and reverse relays. This facilitates the analysis of the distribution of optimal settings across all sample scenarios.
  - The best optimal settings were identified by locating the peaks of the PDFs for the TMS and PS parameters. These settings represent the most frequently occurring optimal configurations across all the sample scenarios.

**Figure 10:**
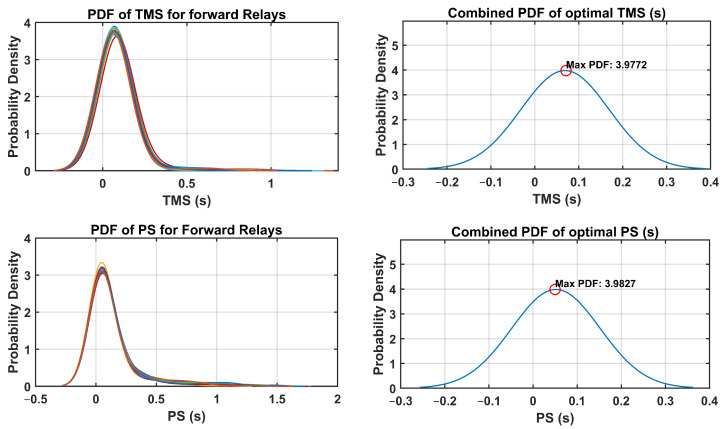
PDF and combined PDF of optimal setting for forward relays in IEEE-14 Bus system.

**Figure 11:**
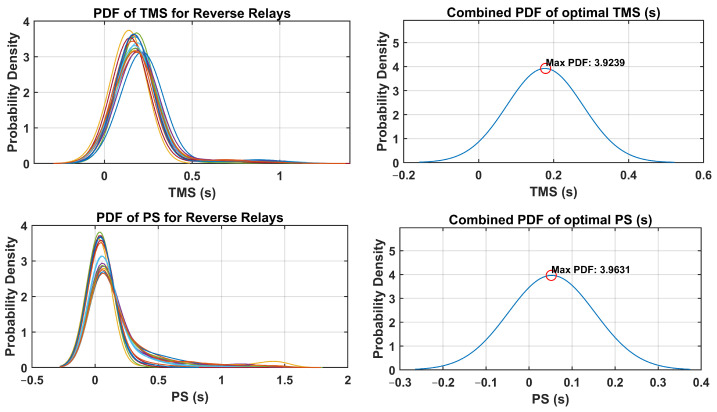
PDF and combined PDF of optimal setting for reverse relays in IEEE-14 Bus system.

The optimal relay settings obtained in this process consider the probabilistic distribution of the fault currents and operating conditions. By integrating uncertainty-aware strategies, the coordination of dual-setting DOCRs is optimized to mitigate the risks associated with potential faults and disturbances in the distribution system. The PDFs of the TMS and PS were analyzed for both forward and reverse relays. Additionally, the combined PDFs of the optimal TMS and PS settings are plotted, highlighting the maximum probability density and their respective optimal settings. These PDFs provide valuable insights into the distribution of the optimal relay settings throughout the network. Through a comprehensive analysis, the optimal TMS and PS values for forward-direction relays were determined, as illustrated in Figure 10. Similarly, Figure 11 presents the optimal TMS and PS values for reverse-direction relays.

**Figure 12:**
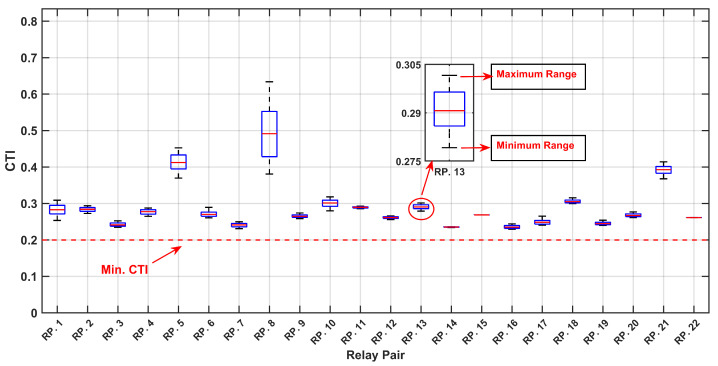
Boxplot of CTI Variation Across MCS Iterations for Relay Pairs, with Optimal Setting Under Uncertainty.

**Figure 13:**
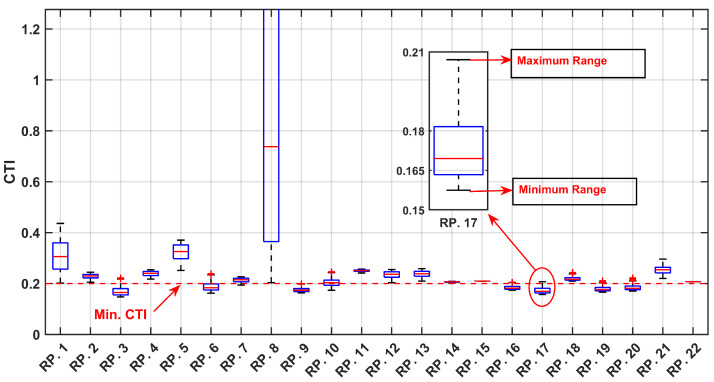
Boxplot of CTI Variation Across MCS Iterations for Relay Pairs, with Optimal Setting without considering uncertainty.

As depicted in Figure 10 and Figure 11 the optimized values for the TMS and PS with the maximum PDF considering uncertainties are notably lower compared to the TMS and PS values without accounting for uncertainties, as indicated in Table 4. This observation underscores the significance of incorporating uncertainties into relay settings, particularly when there are variations in the input data, such as load and generation uncertainties. This highlights the necessity of accounting for uncertainties to ensure a more accurate and reliable relay coordination in the face of varying system conditions. Figure 12 and Figure 13 depict the boxplot graphical representation of the relay operation times in states where uncertainties are considered and not considered, respectively. In Figure 12, all CTI ranges (boxplots) of relay pairs in the state considering uncertainties meet the threshold of $CTI_{min}=0.2$ (highlighted by a red line). However, in Figure 13, relay pairs such as RP.3,RP.6,RP.9,RP.10,RP.16,RP.17,RP.19, and RP.20 do not satisfy the minimum value of $CTI_{min}=0.2$ when uncertainties are not taken into account.

### 4.2. Case 2: IEEE-30 Bus System

**Figure 14:**
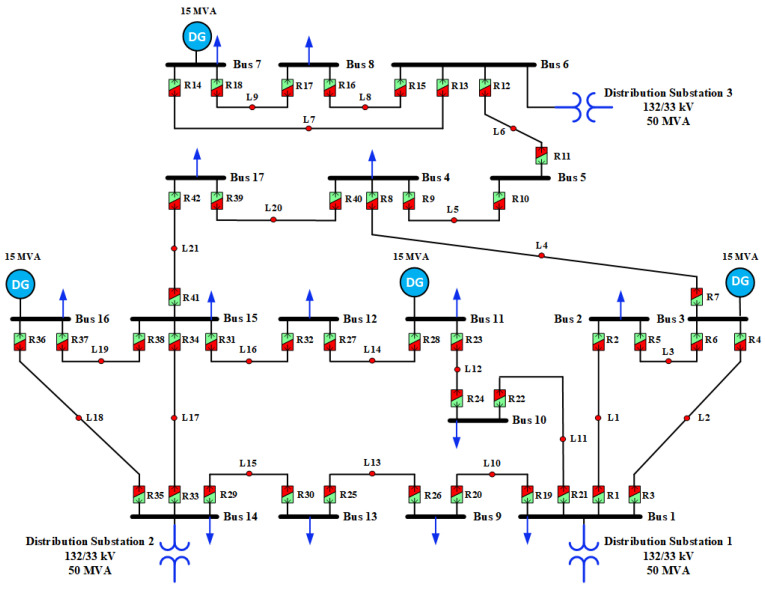
Modified distribution portion (17-Bus Network) of the IEEE-30 Bus system.

To validate the effectiveness of the newly proposed protection strategy, its application was extended from the distribution section of the IEEE-14 bus test system to a larger microgrid setup. Specifically, the novel protection technique was deployed within the distribution segment of the IEEE-30 bus test system, which represents a 17-bus microgrid configuration [46]. This system consists of 21 lines, safeguarded by 42 dual-setting DOCRs positioned at both ends of the lines, providing a more thorough validation of the proposed methodology. Within this setup, four DG sources are integrated, each with a capacity of 15 MVA and connected to buses B3, B7, B11, and B16. Additionally, the system is linked to the utility grid through buses B1, B6, and B14, as illustrated in Figure 14. Detailed information regarding the number of primary and backup relays can be found in Table 5.

In this case study, two scenarios are examined, one in which uncertainties are considered and one in which they are not. The ALO algorithm was utilized to optimize the coordination of dual-setting DOCRs within the test network. Initially, the relay settings were determined without considering the uncertainties. Fault current readings obtained from the P/B relays in the distribution section of the IEEE-30 bus system is shown in Table 6. Furthermore, Table 7 provides information on the operating times of the P/B relay pairs and their corresponding CTI values. The optimal settings for dual-setting DOCRs are presented in Table 8. This analysis aims to evaluate the performance of the proposed protection strategy under different conditions, shedding light on its effectiveness in mitigating faults and enhancing the system reliability.

**Figure 15:**
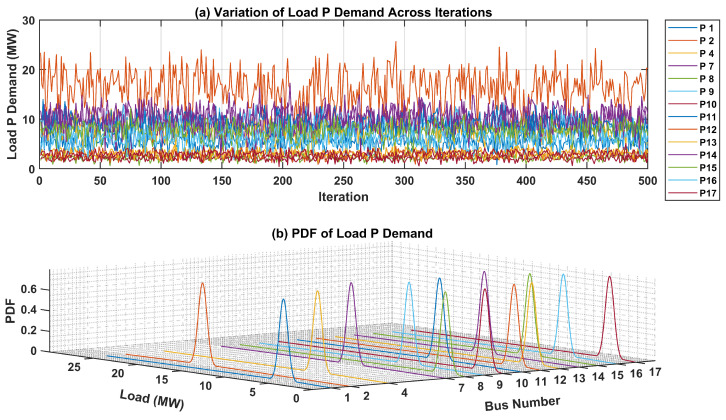
Variation in and PDF of Active Power Load Demand in IEEE-30 Bus system.

**Figure 16:**
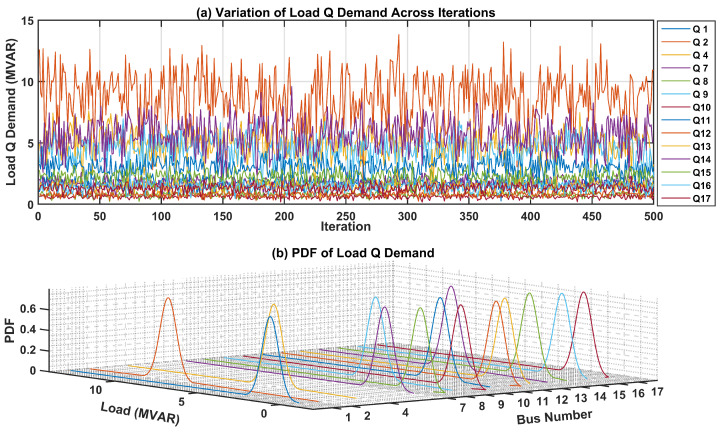
Variation in and PDF of Reactive Power Load Demand in IEEE-30 Bus system.

In the subsequent phase, aimed at assessing and comparing the effectiveness of the proposed protection scheme, particularly in addressing uncertainties about demand load and generation, the fuzzy Monte Carlo technique, as detailed in a previous case study, was employed to analyze the input parameters of the current investigation. To visually depict these uncertainties, PDFs for both active and reactive power of load demands at each bus are presented. Figure 15a illustrates the fluctuation of active power load demand across numerous iterations, offering valuable insights into its temporal variability. Furthermore, the 3D plot in Figure 15b shows the PDF of the active power load demand for each bus. Each curve in this plot represents the PDF of the active power load demand at a specific bus, offering a graphical depiction of the uncertainties associated with the active power load demand. Similarly, Figure 16a illustrates the dynamic fluctuation of reactive power load demand across diverse iterations, offering valuable insights into its temporal variability. Concurrently, the 3D plot in Figure 16b delineates the PDF of the reactive power load demand for each bus within the test network. These visual representations serve to elucidate the inherent uncertainties associated with load demand across various buses within the network, thus enhancing the comprehensiveness and reliability of modeling and analysis.

**Figure 17:**
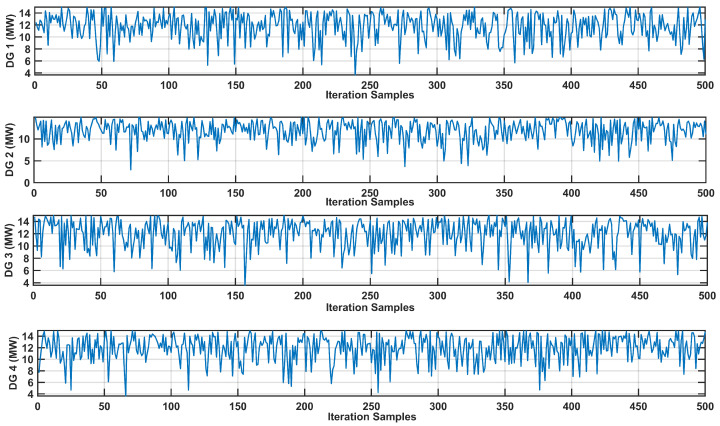
Variation in DG Outputs Across MCS Iterations in IEEE-30 Bus system.

Conversely, uncertainties are also present in the output power of DG sources. Figure 17 depicts the fluctuations in DG source output power across numerous iterations, providing valuable insights into the variability of their power generation. Each subplot in the figure corresponds to the output power of a specific DG source through iterations, effectively highlighting the uncertainties inherent in the DG source power generation. These visual representations offer a comprehensive understanding of the variability in DG source power output, contributing to a more nuanced analysis of the system.

**Figure 18:**
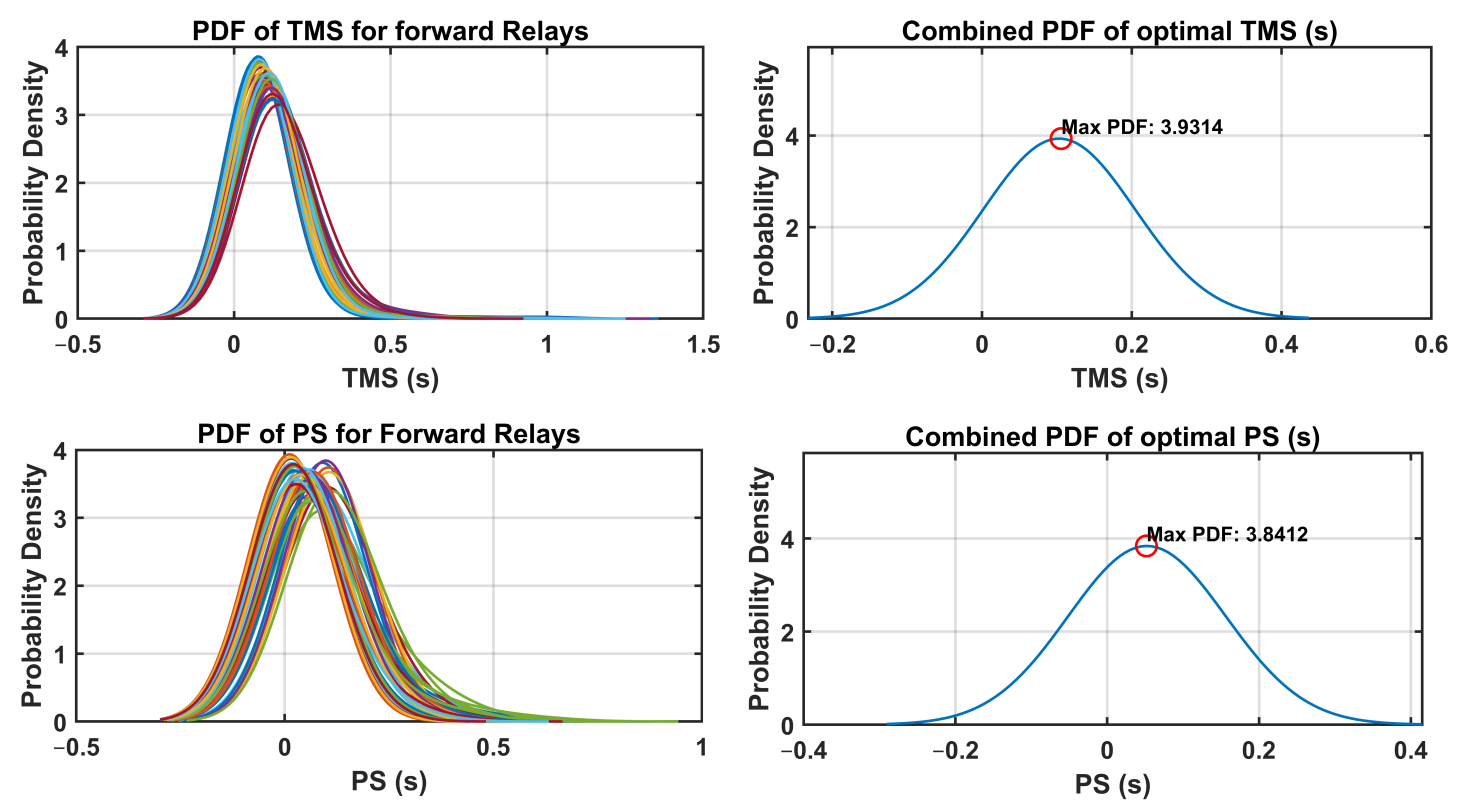
PDF and combined PDF of optimal setting for forward relays in IEEE-30 Bus system.

**Figure 19:**
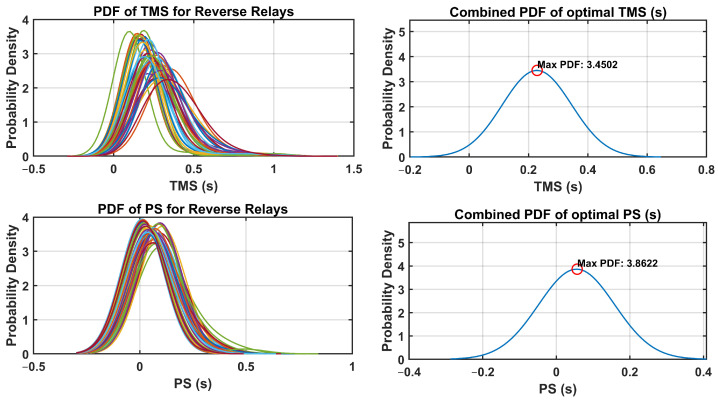
PDF and combined PDF of optimal setting for reverse relays in IEEE-30 Bus system.

The optimal relay settings derived through the fuzzy Monte Carlo technique consider the probabilistic distribution of fault currents and operating conditions. Incorporating strategies that acknowledge the uncertainty, the coordination of dual-setting DOCRs is fine-tuned to mitigate the risks associated with potential faults and disturbances in the distribution system. An analysis of PDFs for TMS and PS was conducted for both forward and reverse relays. Moreover, combined PDFs showing the optimal TMS and PS settings were plotted, highlighting the maximum probability density with their corresponding optimal configurations. These PDFs offer valuable insight into the spread of optimal relay settings across networks. Through examination, the optimal TMS and PS values for forward-direction relays were determined, as depicted in Figure 18. Similarly, Figure 19 illustrates the optimal TMS and PS values for reverse-direction relays.

**Figure 20:**
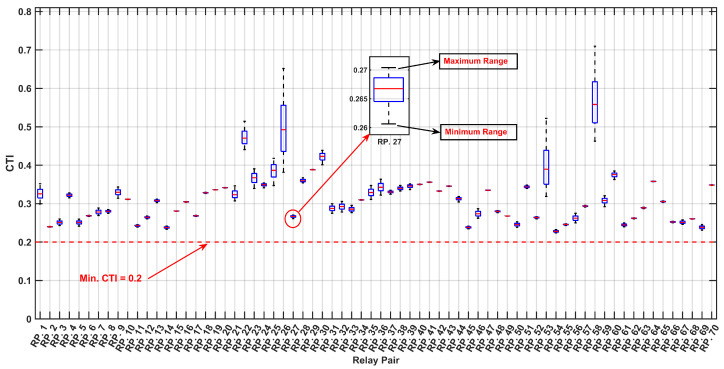
Boxplot of CTI Variation Across MCS Iterations for Relay Pairs, with Optimal Setting Under Uncertainty.

**Figure 21:**
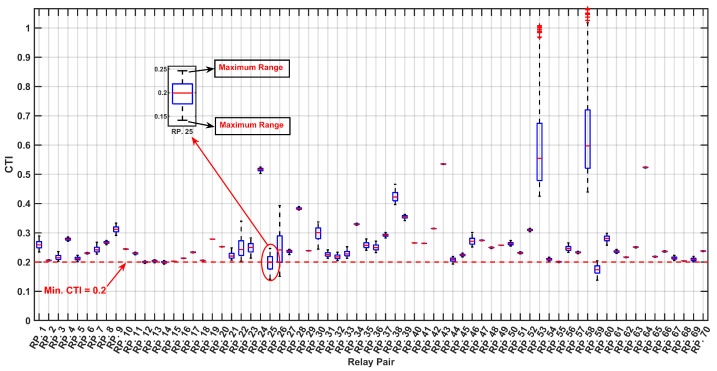
Boxplot of CTI Variation Across MCS Iterations for Relay Pairs, with Optimal Setting without considering uncertainty.

As illustrated in Figure 18 and Figure 19, the optimized values for the TMS and PS maximize the PDF while considering uncertainties, are notably lower than the TMS and PS values when uncertainties are not accounted for, as indicated in Table 8. This observation underscores the critical importance of integrating uncertainties into relay settings, particularly in scenarios where there are fluctuations in the input data, such as load and generation uncertainties. This emphasizes the imperative need to address uncertainties to ensure a more precise and dependable relay coordination amid varying the system conditions. Figure 20 and Figure 21 present boxplot graphical representations of relay operation times in states in which uncertainties are considered and not considered, respectively. In Figure 20, all CTI ranges (boxplots) of relay pairs in the state considering uncertainties fulfill the threshold of $CTI_{min}=0.2$ (highlighted by a red line). However, in Figure 21, relay pairs such as RP.12, RP.13, RP.14, RP.25, RP.26, and RP.59 fail to meet the minimum value of $CTI_{min}=0.2$ when the uncertainties are not factored in.

## 5. Conclusions

In today’s evolving power networks, the integration of distributed generation resources introduces a unique set of challenges in ensuring reliable and effective protection. As discussed in this paper, optimizing the coordination of directional overcurrent relays (DOCRs) become increasingly complex when confronted with uncertainties stemming from both distributed generation (DG) outputs and load consumption patterns. Consequently, this study underscores the importance of integrating uncertainties into the optimization process of dual-setting DOCRs. By employing advanced techniques, such as the fuzzy Monte Carlo method, it is demonstrated that considering uncertainties in relay settings significantly impacts the resulting values of the Time Multiplier Setting (TMS) and Pickup Current Setting (PS). The analysis, as evidenced by the simulation results, indicates that optimized relay settings, when accounting for uncertainties, tend to be notably lower than those obtained without considering uncertainties. This observation highlights the necessity of incorporating uncertainties, especially in environments where variations in input data, such as load and generation uncertainties prevail. The fuzzy Monte Carlo method plays a critical role in this study by simulating the various uncertainties associated with DG and load patterns. These simulations generate a range of possible scenarios, reflecting the real-world variability of power networks. To efficiently handle and optimize these scenarios, the ant lion optimization (ALO) Algorithm is employed. The ALO algorithm is particularly suited for this task due to its robustness in finding optimal solutions in complex, multidimensional search spaces. By integrating the results from the fuzzy Monte Carlo simulations with the ALO algorithm, the study ensures that the optimization process accounts for the inherent uncertainties, thereby enhancing the reliability and accuracy of the relay settings. The findings emphasize the pivotal role of uncertainty-aware strategies in enhancing the accuracy and reliability of relay coordination, thereby ensuring robust protection against potential faults and disturbances in distribution systems. Furthermore, graphical representations illustrate the tangible benefits of accounting for uncertainties in relay operation times. The disparities observed between relay pairs when uncertainties are considered and when they are not underscore the potential risks associated with disregarding uncertainties in relay settings. In essence, this study advocates for a paradigm shift towards embracing uncertainty-aware approaches in the design and optimization of relay settings. By doing so, the power system can better adapt to its dynamic nature, ultimately enhancing its resilience and reliability in the face of uncertain operating conditions.

## Figures and Tables

**Table 1:** The primary/backup protective relays.

Relay Pair	Primary Relay	Backup Relay	Relay Pair	Primary Relay	Backup Relay
RP1	R1	R3	RP12	R8	R11
RP2	R1	R5	RP13	R9	R7
RP3	R2	R12	RP14	R10	R16
RP4	R3	R1	RP15	R11	R8
RP5	R3	R5	RP16	R12	R2
RP6	R4	R13	RP17	R13	R4
RP7	R5	R1	RP18	R14	R15
RP8	R5	R3	RP19	R14	R6
RP9	R6	R14	RP20	R15	R6
RP10	R6	R15	RP21	R15	R14
RP11	R7	R9	RP22	R16	R10

**Table 2:** Fault currents through primary and backup relay pairs.

Primary Relay	Fault Current (A)	Backup Relay	Fault Current (A)	Primary Relay	Fault Current (A)	Backup Relay	Fault Current (A)
R1	10,653.6	R3	1222.4	R8	4713.5	R11	4704.0
R1	10,653.6	R5	2649.6	R9	8183.2	R7	1255.2
R2	7324.3	R12	2555.1	R10	2892.0	R16	3040.1
R3	8858.6	R1	1480.5	R11	6593.3	R8	6593.2
R3	8858.6	R5	1647.9	R12	7922.3	R2	3363.8
R4	6079.3	R13	2093.6	R13	6530.3	R4	2051.0
R5	13,330.2	R1	2246.6	R14	7981.2	R15	2656.4
R5	13,330.2	R3	974.1	R14	7981.2	R6	940.0
R6	9472.2	R14	1877.4	R15	6729.0	R6	2025.4
R6	9472.2	R15	1413.2	R15	6729.0	R14	933.4
R7	14,970.6	R9	1444.0	R16	3232.1	R10	3074.6

**Table 3:** Operational times between P/B relay pairs with CTI.

Relay Pairs		ALO			Relay Pairs		ALO		
Primary	Backup	Tp (s)	Tb (s)	CTI (s)	Primary	Backup	Tp (s)	Tb (s)	CTI (s)
R1	R3	0.101	0.302	0.201	R8	R11	0.106	0.309	0.204
R1	R5	0.101	0.302	0.201	R9	R7	0.104	0.314	0.210
R2	R12	0.141	0.347	0.206	R10	R16	0.104	0.309	0.205
R3	R1	0.128	0.342	0.213	R11	R8	0.103	0.307	0.205
R3	R5	0.128	0.354	0.226	R12	R2	0.109	0.310	0.202
R4	R13	0.116	0.328	0.212	R13	R4	0.103	0.319	0.216
R5	R1	0.103	0.304	0.201	R14	R15	0.103	0.358	0.255
R5	R3	0.103	0.329	0.226	R14	R6	0.103	0.304	0.200
R6	R14	0.101	0.302	0.201	R15	R6	0.117	0.325	0.208
R6	R15	0.101	0.301	0.200	R15	R14	0.117	0.370	0.253
R7	R9	0.105	0.313	0.208	R16	R10	0.103	0.307	0.204

**Table 4:** Optimal setting for dual-setting DOCRs in distribution part of the IEEE-14 Bus System using ALO.

Relay No.	Forward		Reverse		Relay No.	Forward		Reverse	
	TMS (s)	PS (pu)	TMS (s)	PS (pu)		TMS (s)	PS (pu)	TMS (s)	PS (pu)
R1	0.074	0.045	0.178	0.025	R9	0.078	0.032	0.162	0.025
R2	0.113	0.021	0.148	0.075	R10	0.041	0.109	0.199	0.023
R3	0.102	0.026	0.125	0.042	R11	0.080	0.021	0.099	0.302
R4	0.082	0.031	0.167	0.034	R12	0.088	0.021	0.157	0.068
R5	0.082	0.038	0.149	0.054	R13	0.070	0.040	0.157	0.047
R6	0.079	0.029	0.193	0.022	R14	0.063	0.077	0.174	0.022
R7	0.094	0.023	0.164	0.021	R15	0.087	0.027	0.116	0.059
R8	0.054	0.085	0.214	0.036	R16	0.040	0.134	0.191	0.028
**OF (s) = 1.747 s**									

**Table 5:** The primary/backup protective relays.

Relay Pair	Primary Relay	Backup Relay	Relay Pair	Primary Relay	Backup Relay	Relay Pair	Primary Relay	Backup Relay
RP1	R1	R3	RP25	R14	R18	RP49	R31	R38
RP2	R1	R19	RP26	R15	R12	RP50	R31	R41
RP3	R1	R21	RP27	R15	R13	RP51	R32	R27
RP4	R2	R5	RP28	R16	R17	RP52	R33	R29
RP5	R3	R1	RP29	R17	R16	RP53	R33	R35
RP6	R3	R19	RP30	R18	R14	RP54	R34	R31
RP7	R3	R21	RP31	R19	R1	RP55	R34	R38
RP8	R4	R6	RP32	R19	R3	RP56	R34	R41
RP9	R4	R7	RP33	R19	R21	RP57	R35	R29
RP10	R5	R2	RP34	R20	R26	RP58	R35	R33
RP11	R6	R4	RP35	R21	R1	RP59	R36	R37
RP12	R6	R7	RP36	R21	R3	RP60	R37	R36
RP13	R7	R4	RP37	R21	R19	RP61	R38	R31
RP14	R7	R6	RP38	R22	R24	RP62	R38	R34
RP15	R8	R9	RP39	R23	R28	RP63	R38	R41
RP16	R8	R40	RP40	R24	R22	RP64	R39	R42
RP17	R9	R8	RP41	R25	R30	RP65	R40	R8
RP18	R9	R40	RP42	R26	R20	RP66	R40	R9
RP19	R10	R11	RP43	R27	R32	RP67	R41	R31
RP20	R11	R10	RP44	R28	R23	RP68	R41	R34
RP21	R12	R13	RP45	R29	R33	RP69	R41	R38
RP22	R12	R15	RP46	R29	R35	RP70	R42	R39
RP23	R13	R12	RP47	R30	R25			
RP24	R13	R15	RP48	R31	R34			

**Table 6:** Fault currents through primary and backup relay pairs.

Primary Relay	Fault Current (A)	Backup Relay	Fault Current (A)	Primary Relay	Fault Current (A)	Backup Relay	Fault Current (A)
R1	11,648.9	R3	670.7	R21	8446.1	R3	829.0
R1	11,648.9	R19	1839.0	R21	8446.1	R19	1008.5
R1	11,648.9	R21	1954.9	R22	3676.2	R24	3675.5
R2	5493.0	R5	5475.9	R23	5633.3	R28	2643.6
R3	8454.8	R1	1771.5	R24	4773.9	R22	4772.4
R3	8454.8	R19	1516.9	R25	4048.0	R30	4049.4
R3	8454.8	R21	1613.0	R26	6137.9	R20	6140.3
R4	6141.8	R6	1777.1	R27	3572.0	R32	3567.2
R4	6141.8	R7	1962.6	R28	5828.0	R23	3212.8
R5	7080.3	R2	7059.4	R29	8237.6	R33	1484.9
R6	9107.0	R4	3339.6	R29	8237.6	R35	983.7
R6	9107.0	R7	2640.4	R30	3428.9	R25	3426.2
R7	7561.2	R4	2217.5	R31	6177.5	R34	3560.5
R7	7561.2	R6	3389.4	R31	6177.5	R38	1519.4
R8	3677.8	R9	1865.3	R31	6177.5	R41	1128.8
R8	3677.8	R40	1806.4	R32	3462.9	R27	3464.2
R9	4173.4	R8	2826.2	R33	9327.5	R29	1477.9
R9	4173.4	R40	1348.9	R33	9327.5	R35	499.0
R10	3220.9	R11	3224.8	R34	5175.9	R31	2035.3
R11	2409.6	R10	2407.2	R34	5175.9	R38	1533.1
R12	7288.1	R13	630.8	R34	5175.9	R41	1605.4
R12	7288.1	R15	437.4	R35	6880.3	R29	1154.0
R13	3670.6	R12	602.9	R35	6880.3	R33	347.3
R13	3670.6	R15	457.7	R36	3876.2	R37	1603.8
R14	2386.1	R18	456.5	R37	4450.0	R36	2095.1
R15	4879.3	R12	732.7	R38	5488.5	R31	1509.8
R15	4879.3	R13	81.5	R38	5488.5	R34	2801.4
R16	1467.2	R17	1466.0	R38	5488.5	R41	1196.5
R17	1937.1	R16	1936.4	R39	3238.3	R42	3237.9
R18	2961.1	R14	963.7	R40	4514.0	R8	3056.0
R19	12,808.9	R1	1957.7	R40	4514.0	R9	1464.7
R19	12,808.9	R3	1279.3	R41	6597.0	R31	1478.5
R19	12,808.9	R21	1756.7	R41	6597.0	R34	3613.4
R20	2892.6	R26	2878.5	R41	6597.0	R38	1537.6
R21	8446.1	R1	1266.0	R42	2596.3	R39	2592.7

**Table 7:** Operational times between P/B relay pairs with CTI.

Relay Pairs		ALO			Relay Pairs		ALO		
Primary	Backup	Tp (s)	Tb (s)	CTI (s)	Primary	Backup	Tp (s)	Tb (s)	CTI (s)
R1	R4	0.130	0.463	0.332	R21	R4	0.168	0.375	0.206
R1	R20	0.130	0.381	0.251	R21	R20	0.168	0.382	0.213
R1	R22	0.130	0.372	0.242	R22	R23	0.113	0.440	0.327
R2	R6	0.188	0.467	0.279	R23	R27	0.116	0.551	0.435
R3	R2	0.119	0.369	0.250	R24	R21	0.104	0.497	0.393
R3	R20	0.119	0.408	0.289	R25	R29	0.316	0.521	0.205
R3	R22	0.119	0.396	0.277	R26	R19	0.134	0.357	0.223
R4	R5	0.177	0.520	0.343	R27	R31	0.101	0.371	0.270
R4	R8	0.177	0.492	0.315	R28	R24	0.284	0.501	0.216
R5	R1	0.180	0.499	0.318	R29	R34	0.108	0.316	0.208
R6	R3	0.153	0.421	0.268	R29	R36	0.108	0.383	0.275
R6	R8	0.153	0.439	0.286	R30	R26	0.116	0.732	0.616
R7	R3	0.158	0.476	0.318	R31	R33	0.112	0.345	0.233
R7	R5	0.158	0.413	0.255	R31	R37	0.112	0.332	0.220
R8	R10	0.149	0.405	0.256	R31	R42	0.112	0.343	0.231
R8	R39	0.149	0.425	0.277	R32	R28	0.138	0.392	0.254
R9	R7	0.174	0.456	0.282	R33	R30	0.116	0.316	0.200
R9	R39	0.174	0.466	0.292	R33	R36	0.116	0.515	0.399
R10	R12	0.194	0.520	0.326	R34	R32	0.100	0.301	0.201
R11	R9	0.178	0.504	0.327	R34	R37	0.100	0.331	0.231
R12	R14	0.147	0.486	0.339	R34	R42	0.100	0.318	0.218
R12	R16	0.147	0.516	0.368	R35	R30	0.131	0.348	0.217
R13	R11	0.173	0.467	0.293	R35	R34	0.131	0.521	0.389
R13	R16	0.173	0.506	0.333	R36	R38	0.212	0.523	0.312
R14	R17	0.199	0.627	0.428	R37	R35	0.120	0.434	0.314
R15	R11	0.163	0.411	0.248	R38	R32	0.123	0.327	0.204
R15	R14	0.163	1.316	1.154	R38	R33	0.123	0.367	0.244
R16	R18	0.175	0.590	0.415	R38	R42	0.123	0.339	0.216
R17	R15	0.191	0.516	0.325	R39	R41	0.151	0.356	0.205
R18	R13	0.182	0.569	0.387	R40	R7	0.218	0.443	0.225
R19	R2	0.126	0.356	0.231	R40	R10	0.218	0.421	0.203
R19	R4	0.126	0.357	0.231	R41	R32	0.119	0.329	0.210
R19	R22	0.126	0.385	0.260	R41	R33	0.119	0.344	0.225
R20	R25	0.186	0.480	0.294	R41	R37	0.119	0.331	0.212
R21	R2	0.156	0.420	0.264	R42	R40	0.113	0.359	0.245

**Table 8:** Optimal setting for dual-setting DOCRs in distribution part of the IEEE-30 bus system using ALO.

Relay No.	Forward		Reverse		Relay No.	Forward		Reverse	
	TMS (s)	PS (pu)	TMS (s)	PS (pu)		TMS (s)	PS (pu)	TMS (s)	PS (pu)
R1	0.076	0.055	0.079	0.209	R22	0.040	0.188	0.343	0.027
R2	0.130	0.038	0.505	0.067	R23	0.038	0.338	0.226	0.086
R3	0.091	0.026	0.162	0.025	R24	0.071	0.029	0.205	0.090
R4	0.050	0.124	0.242	0.041	R25	0.204	0.030	0.445	0.033
R5	0.193	0.071	0.154	0.238	R26	0.095	0.031	0.252	0.041
R6	0.060	0.133	0.170	0.108	R27	0.072	0.018	0.151	0.144
R7	0.120	0.060	0.151	0.064	R28	0.196	0.033	0.229	0.089
R8	0.055	0.105	0.199	0.083	R29	0.056	0.145	0.128	0.054
R9	0.045	0.113	0.144	0.080	R30	0.043	0.159	0.255	0.084
R10	0.129	0.085	0.145	0.129	R31	0.081	0.028	0.176	0.023
R11	0.050	0.154	0.287	0.094	R32	0.094	0.021	0.250	0.022
R12	0.059	0.084	0.099	0.070	R33	0.082	0.047	0.178	0.019
R13	0.103	0.005	0.239	0.003	R34	0.063	0.043	0.218	0.029
R14	0.078	0.030	0.122	0.055	R35	0.090	0.041	0.152	0.037
R15	0.072	0.091	0.238	0.004	R36	0.098	0.096	0.343	0.006
R16	0.047	0.039	0.157	0.055	R37	0.075	0.039	0.316	0.016
R17	0.058	0.026	0.374	0.048	R38	0.093	0.020	0.215	0.011
R18	0.134	0.003	0.181	0.008	R39	0.119	0.010	0.294	0.006
R19	0.078	0.098	0.139	0.048	R40	0.173	0.013	0.203	0.019
R20	0.060	0.030	0.234	0.044	R41	0.087	0.030	0.246	0.005
R21	0.115	0.051	0.158	0.040	R42	0.090	0.008	0.301	0.007
**OF (s) = 6.084 s**									

## Data Availability

Data are contained within the article.

## Abbreviations

The following abbreviations are used in this manuscript:

ADN	Active distribution networks.
ALO	Ant lion optimizer algorithm.
CTI	Coordination time interval.
DOCRs	Directional overcurrent relays.
DG	Distributed generation.
DS-DOCRs	Dual-setting directional overcurrent relays.
MCS	Monte Carlo simulation.
MGs	Microgrids.
OLF	Overload factor.
OCR	Overcurrent relay.
PS	pickup (plug) setting.
PFMC	Probabilistic model of fuzzy Monte Carlo.
PDF	Probability density function.
P/B	Primary/backup.
SGs	Smart grids.
TMS	Time multiplier setting.
